# Complete Genome Sequence of Agrobacterium tumefaciens 1D1609

**DOI:** 10.1128/genomeA.00253-18

**Published:** 2018-04-19

**Authors:** Shu-Ting Cho, Mindia Haryono, Hsing-Hua Chang, Mary Nia M. Santos, Erh-Min Lai, Chih-Horng Kuo

**Affiliations:** a Institute of Plant and Microbial Biology, Academia Sinica, Taipei, Taiwan

## Abstract

Agrobacterium tumefaciens 1D1609 is a highly virulent strain isolated from a crown gall tumor of alfalfa (Medicago sativa L.). Compared to other well-characterized A. tumefaciens strains, such as C58 and Ach5, 1D1609 has a distinctive host range. Here, we report its complete genome sequence to facilitate future studies.

## GENOME ANNOUNCEMENT

Agrobacterium tumefaciens is known for its ability to transfer a DNA segment on the tumor-inducing (Ti) plasmid into the nuclear genome of its plant hosts (1). Because of this property, A. tumefaciens is widely used in genetic engineering (2, 3). The strain A. tumefaciens 1D1609 was isolated from a crown gall on a field-grown alfalfa plant in Imperial Valley, southern California (4). Previous infection assays demonstrated that this strain has an infectivity profile distinct from those of other well-characterized strains, such as C58 and Ach5 (4, 5). Thus, comparative analysis among these A. tumefaciens strains could shed light on the genetic determinants of host range and infection efficiency, which would improve their biotechnological applications. To facilitate such studies, we determined the complete genome sequence of A. tumefaciens 1D1609 and report the results here.

The procedures for sequencing, assembly, and annotation are based on those described previously (6–8). Briefly, the Illumina MiSeq platform was used to generate 301-bp reads from one paired-end library (∼550-bp insert, 11,564,340 reads) and one mate pair library (∼4,100-bp insert, 8,219,766 reads). The *de novo* assembly was performed using AllPaths-LG (9). The initial draft assembly was iteratively improved using PAGIT (10). In each iteration, the Illumina reads were mapped to the assembly using Burrows-Wheeler Aligner (BWA) (11), programmatically checked using SAMtools (12), and visually inspected using Integrative Genomics Viewer (IGV) (13). The regions with repetitive sequences (e.g., rRNA gene clusters) or low coverage of Illumina reads were confirmed by PCR and Sanger sequencing. The iterative process was continued until the complete genome assembly was obtained and verified. Gene prediction was done using RNAmmer (14), tRNAscan-SE (15), Prodigal (16), and GeneMark.hmm (17). The initial annotation was based on the homologous genes in A. tumefaciens C58 (18–20) and Ach5 (8) as identified by OrthoMCL (21). Subsequently, manual curation was performed based on BLASTP (22) searches against the National Center for Biotechnology Information (NCBI) nonredundant protein database (23), the NCBI Conserved Domain Database (CDD) (24), and the Kyoto Encyclopedia of Genes and Genomes (KEGG) database (25, 26). Finally, noncoding RNAs were annotated based on the Rfam database (27).

The complete genome sequence of A. tumefaciens 1D1609 consists of one circular chromosome (3,058,772 bp), one linear chromosome (2,329,227 bp), one octopine-type Ti plasmid (166,117 bp), and two other plasmids (pAt1D1609a, 243,381 bp; pAt1D1609b, 187,640 bp). The first version of annotation includes 12 rRNA genes, 53 tRNA genes, 5,630 protein-coding genes, and 15 noncoding RNAs.

### Accession number(s).

The complete genome sequence of A. tumefaciens 1D1609 has been deposited at DDBJ/EMBL/GenBank under the accession numbers CP026924 to CP026928.
